# If Gaming is the Problem, Is "Complexity Thinking" the Answer? A Response to the Recent Commentaries

**DOI:** 10.34172/ijhpm.2020.95

**Published:** 2020-06-20

**Authors:** Tim Tenbensel, Peter Jones, Linda Chalmers, Shanthi Ameratunga, Peter Carswell

**Affiliations:** ^1^School of Population Health, University of Auckland, Auckland, New Zealand.; ^2^Auckland District Health Board, University of Auckland, Auckland, New Zealand.; ^3^Wintec, Hamilton, New Zealand.

We would like to thank the authors of each of the three commentaries^1-3^ for their very useful and thought-provoking comments on our article on the gaming of New Zealand’s emergency department (ED) target.^4^ We were heartened by Lisa M Lines’ comment that ‘performance measure developers, healthcare providers and administrators, policy-makers, and researchers in the field would do well to be both humbled and encouraged by this research.’^3^ The key findings of our broader research programme into New Zealand’s ED target implementation^4-9^ that are pertinent to the broader literature on performance measurement and management in healthcare are:

- Zealand’s ED target induced an initially positive response that prompted important system changes that are likely to have saved hundreds of lives.
- Over time, the capacity for innovation reached limits, and in the face of increasing utilisation of ED services, subsequent achievement of the target was only possible through gaming and other dysfunctional consequences.
- These consequences unfolded differently, and generated different responses across implementation settings.

All three commentaries prompt wider reflection on the implications of our findings about the design and implementation of performance measurement in healthcare. Rather than responding individually to each commentary, we have picked out two common themes. All commentaries situate performance management and gaming in the context of complexity which is key to understanding the potential and the problems of performance measurement. Consequently, two of the commentaries^1,2^ advocate developing a more trust-based and less hierarchical approach to performance measurement and management.

We agree with all commentaries that thinking of EDs and hospitals in terms of complexity and complex adaptive systems can lead to a more sophisticated approach to performance management. Our research showed that implementers based in EDs had some scope to influence ED wait times, particularly around patient flow processes. However, after around eighteen months, further improvements in ED waiting times were dependent on other parts of the system that they could not control. Most important among these were the practices of inpatient specialists elsewhere in the hospitals acting as gatekeepers to the wards. The use of short stay units as holding bays for potential target breaches can be clearly seen as an emergent phenomenon in response to the twin stimuli of the target and gatekeeping on the wards.

In the light of this complexity-inspired interpretation, Chen makes further recommendations,^1^ which strongly resonate with the perspective of Hamblin and Shuker.^2^ Both commentaries advocate an approach to performance measurement that is driven more by the input and sense-making capacity of frontline clinicians and managers, and trust in their intrinsic motivation and interest in quality, and less by the imperatives of external sanctions and accountability to the government of the day.

Overall, our research into ED target implementation supports Hamblin and Shuker’s^2^ and Chen’s^1^ preferred approach. While senior ED clinicians and managers were supportive of the target, much of the burden of implementation (and the impetus for gaming) was felt most strongly by others such as ED nurses. Also, the definition of the performance measure as ‘ED’s target’ served to minimise buy-in from clinicians outside the ED. A complex adaptive systems perspective would certainly help policy-makers anticipate these reactions.^10,11^

Secondly, both Chen and Hamblin and Shuker justifiably take issue with our recommendation that more effective audit can mitigate the extent and effects of gaming. They do so partly on grounds of costs and resources, but also because audit would accentuate the top-down dynamics of the target, rather than fostering more collaborative ownership of performance improvement. Their recommendations are also broadly consistent with recent developments in public management literature, particularly from Van Dooren and Hoffmann^12^ that performance management works best when linked to the purpose of learning and improvement, rather than accountability.

While we broadly agree that the next generation of approaches to performance management needs to build on intrinsic motivations and interests of front-line staff, there are some important limitations to relying on a decentralised, quality-improvement approach. Our research has shown that local, organisational context matters. Any development of a more ‘bottom-up,’ learning approach to performance improvement, therefore, is likely to have variable impact, and is likely to be dependent on pre-existing levels of trust within and between organisations. If, as Chen argues, ‘we need to build relationship, maintain dialogue, mutually respect each profession’s norm and value, and muddle through’^1^ the results are likely to vary according to the willingness of practitioners to cede or share some control over their professional domain. This may well produce a very uneven pattern of performance and learning^13^ unless there are well-developed and powerful mechanisms for spreading good practice that can overcome institutional barriers in *every* location.

This brings us back to governmental stewardship of health systems. The New Zealand’s ED target was part of regime of six targets which were the cornerstone of the government’s health policy strategy. Arguably, the issue of ED crowding and excessive waiting times would not have been addressed if it had not been elevated to a high-level policy priority. New Zealand’s tax-funded health system sheets home responsibility for health service problems directly to governments. In applying a complexity lens, this broad context of democratic accountability needs to be taken seriously as an important feature of the system, rather than regarded as an artificial impediment to the capacity of systems to harness the potential of complex adaptive systems.^14^ In such contexts, energy for change is likely to come from both the ‘bottom’ and the ‘top.’

For those with responsibilities for developing health system and health service performance measurement regimes, we recommend adopting a way of thinking about system change suggested by Kreindler.^15^ This involves fostering a dynamic relationship between processes of ‘stipulation’ (precisely defining a specific response) and ‘stimulation’ (building on behaviour already present or emerging within the system). This approach is inspired by complexity thinking, and emphasises the need for continual adaptation and evolution of performance management approaches. Our research into gaming has shown what can happen when this adaptation at the policy level is absent.

## Ethical issues

Not applicable.

## Competing interests

Authors declare that they have no competing interests.

## Authors’ contributions

TT drafted the commentary and all other authors were given the opportunity to comment on it before submission.

## Authors’ affiliations

^1^School of Population Health, University of Auckland, Auckland, New Zealand. ^2^Auckland District Health Board, University of Auckland, Auckland, New Zealand. ^3^Wintec, Hamilton, New Zealand.
